# Levels and Distribution of Pollutants in the Waters of an Aquatic Ecosystem in Northern Mexico

**DOI:** 10.3390/ijerph14050456

**Published:** 2017-04-25

**Authors:** Jesús Manuel Ochoa-Rivero, Ana Victoria Reyes-Fierro, Ma. Del Rosario Peralta-Pérez, Francisco Javier Zavala-Díaz de la Serna, Lourdes Ballinas-Casarrubias, Ivan Salmerón, Héctor Rubio-Arias, Beatriz A. Rocha-Gutiérrez

**Affiliations:** 1 Sitio Experimental la Campana, CIRNOC, Instituto Nacional de Investigaciones Forestales, Agrícolas y Pecuarias (INIFAP), Km. 33.3 Carretera Chihuahua-Ojinaga. Aldama, Chihuahua C.P. 32910, Mexico; jochoarivero@gmail.com; 2 Facultad de Ciencias Químicas, Universidad Autónoma de Chihuahua, Campus Universitario #2, Circuito Universitario, Chihuahua, Chihuahua C.P. 31125, Mexico; viickyr27@gmail.com (A.V.R.-F.); rosariopp16@yahoo.com.mx (M.D.R.P.-P.); javierzavala@hotmail.com (F.J.Z.-D.d.l.S.); lourdes.ballinas@gmail.com (L.B.-C.); isalmeron@uach.mx (I.S.); 3 Facultad de Zootecnia y Ecología, Universidad Autónoma de Chihuahua, Periférico. R. Almada, km. 1. Chihuahua, Chihuahua C.P. 31453, Mexico; rubioa1105@hotmail.com

**Keywords:** DDT, agriculture, pesticides, water, sodium adsorption ratio (SAR), wastewater, water pollution

## Abstract

The availability of good quality water resources is essential to ensure healthy crops and livestock. The objective of this study was to evaluate the level of pollution in Bustillos Lagoon in northern Mexico. Physical-chemical parameters like sodium, chloride, sulfate, electrical conductivity, nitrates, and the pesticide dichlorodiphenyltrichloroethane (DDT) were analyzed to determine the water quality available in the lagoon. Although DDT has been banned in several countries, it is still used for agricultural purposes in Mexico and its presence in this area had not been analyzed previously. Bustillos Lagoon was divided into three zones for the evaluation: (1) industrial; (2) communal lands; and (3) agricultural. The highest concentrations of sodium (2360 mg/L) and SAR (41 meq/L) reported in the industrial zone are values exceeding the United Nations Food and Agricultural Organization (FAO) irrigation water quality guidelines. DDT and its metabolites were detected in all of the 21 sites analyzed, in the agricultural zone ∑DDTs = 2804 ng/mL, this level is much higher than those reported for other water bodies in Mexico and around the world where DDT has been used heavily. The water in the communal zone is the least contaminated, but can only be recommended for irrigation of plants with high stress tolerance and not for crops.

## 1. Introduction

Water scarcity is a major problem facing many societies around the world. Available water resources can become polluted as a consequence of anthropogenic activities like ongoing untreated wastewater discharges, the ingress of dangerous chemicals caused by irrigation runoff and overexploitation of aquifers [1,2].

The use of wastewater for irrigation is increasingly being practiced around the world. In Mexico, as in other developing countries, wastewater is often used for crop irrigation. It is estimated that about 190,000 ha in Mexico are irrigated with untreated wastewater [3].

According to the United Nations Food and Agricultural Organization (FAO), parameters that affect water quality include high concentrations of ions like chloride, sodium, sulfate, and magnesium, which can reduce crop and livestock production, quality, and health [4]. Other chemicals that can pollute water are organochlorine pesticides (OCPs).

OCPs were used extensively around the world in agriculture and malaria control from 1940 to 1960. Many organochlorine pesticides were banned in the United States and other countries in the 70’s due to reported toxicological effects. A representative compound of this group is the pesticide dichlorodiphenyltrichloroethane (DDT), which was the most widely used in agriculture worldwide from 1946 until 1972 until it was considered too hazardous owing to its toxicity, stability in the environment and high lipophilicity [5,6,7]. A major concern about DDT is its capacity to bioaccumulate in the food chain and thus threatens both human health and the environment. Studies with male and female rats have reported that DDT is an endocrine disrupter due to its adverse effects on reproductive organs [8]. Exposure of the uterus to DDT increases the risk of breast cancer [9]. The principle route of human exposure to DDT is consumption of foods contaminated by irrigation with waters containing trace concentrations of this contaminant [10,11].

The use of DDT in Mexico began in the 1940s. Between the mid-1950s to 1999 approximately 70,000 tons of DDTs were applied to control the malaria mosquito and for agricultural purposes [12]. In 2005, Mexico was ranked fourth for the highest agricultural use of DDT during the period 1948–2000, applying 180 kT of DDT. It also was ranked fifth for use in public health programs [5]. Currently, there are limits on the use of DDT in Mexico, but it is suspected that it is still used in agriculture because of its effectiveness and affordability [13]. DDT is banned in 59 countries and its use is severely restricted in 20 (among them Mexico) [5]. Recent studies of water bodies in Mexico have reported high levels of DDT and its metabolites: DDE (dichlorodiphenyldichloroethylene) and DDD (dichlorodiphenyldichloroethane) [14,15,16,17,18].

Bustillos Lagoon has been considered a priority wetland by the US Fish and Wildlife Service (USFWS) [19]. The Commission for Environmental Cooperation (CEC) listed this lagoon among the 150 priority hydrological regions in the Directory of North American Important Bird Areas (IBA) due to its biodiversity, as well as one of the most threatened ecosystems because of the anthropogenic activities [20]. The Mexican Institution, the Comisión Nacional para el Conocimiento y Uso de la Biodiversidad (CONABIO), considered this lagoon an important ecosystem in Mexico for the 186 birds species recorded in this area [21].

Although there have been a few studies of the quality of water in Bustillos Lagoon, none have reported on the levels of organochlorine pesticides like DDT and its metabolites [22,23,24,25]. The main discharges to Bustillos Lagoon are runoff from an area of intensive agriculture, and industrial and domestic wastewater discharges [18]. Water from the lagoon is used for crop irrigation and other agricultural applications [24,25].

Thus, this study evaluated the physical-chemical parameters that determine current water quality and DDT exposure, which can represent a high risk for the ecosystem and human health if the water is used for crop irrigation and as livestock drinking water.

The aims of this work were to: (1) evaluate the water quality of Bustillos Lagoon based on the physical-chemical parameters; (2) investigate the presence and distribution of DDT and its metabolites in the lagoon; and (3) determine if the water is safe for irrigation and/or as livestock drinking water.

## 2. Materials and Methods

### 2.1. Study Area

**Figure 1:**
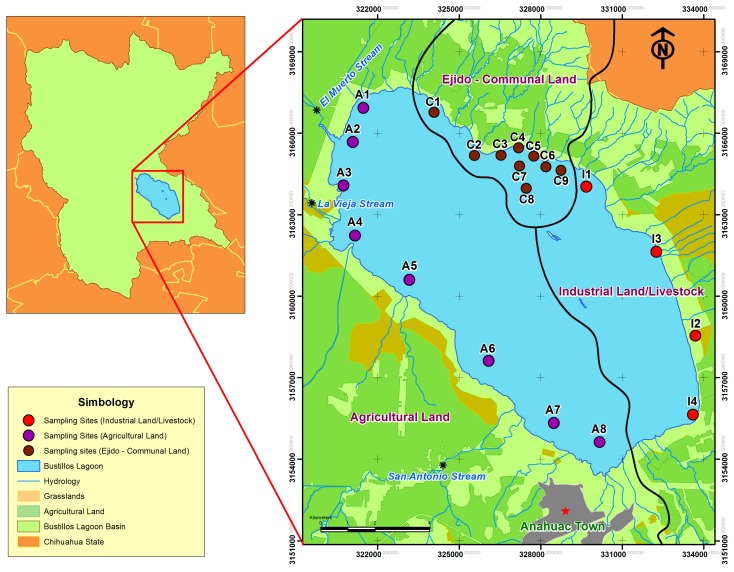
Location of Bustillos Lagoon in Chihuahua, Mexico and sampling sites. Sample codes I = Industrial (Zone 1), C = Communal land (Zone 2), A = Agricultural land (Zone 3).

The study was carried out in the endorheic basin called Bustillos Lagoon, which forms part of the second most extensive hydrological region in the state of Chihuahua, Mexico. The study area was divided into three zones (clusters) using the geographic information system Google™ Earth for the evaluation: industrial (zone 1), communal lands (zone 2) and agricultural (zone 3), as shown in Figure 1. Agricultural land is defined as an area with intensive agriculture. Communal land refers to a community where the law establishes that any timber or grazing lands must be used collectively or shared. This has resulted in most communal areas being in poor condition.

The lagoon is located between latitude 28°33’32’’ N and longitude 106°45’57’’ W, at an altitude of 1975.5 m. It covers an area of approximately 107 km^2^. The main streams that feed Bustillos Lagoon are: El Muerto, La Quemada, Santa Elena, Bustillos, San Antonio, La Vieja and Napavechi, as well as several other smaller influent streams [26,27]. The main activities in the surrounding areas are agriculture, raising livestock, and dairy farming. The crops cultivated in the area include maize, wheat, barley, oat, grasses, apple and vegetables [28,29,30]. Organophosphate pesticides like malathion, parathion and 2,4-D amine are commonly used to improve crop yields [25], but it is uncertain if organochlorine pesticides such as DDTs are still being applied.

### 2.2. Materials and Reagents

Organochlorine pesticide mixtures containing 2,4’-DDD, 4,4’-DDD, 2,4’-DDE, 4,4’-DDE, 2,4’-DDT, and 4,4’-DDT at 50 µg/mL in acetone was supplied by ULTRA Scientific (North Kingstown, RI, USA). The internal standard 1-bromine, 2-nitrobenzene at 1000 µg/mL in acetone was supplied by AccuStandard (New Haven, CT, USA). The Acetone used was chromatography grade (J.T. Baker). Ultrapure water was obtained from a FESTA ULTRA-pure water production system (model UP 09 99 2017) (Chihuahua, Mexico). The manual SPME holders and 100 µm polydimethylsiloxane (PDMS) (fibers were purchased from Suppelco (St. Louis, MO, USA). Prior to use, SPME fibers were conditioned as recommended by manufacturer. Analytical reagents were used for ion chromatography (IC) analysis.

### 2.3. Water Sample Collection

Water samples were collected after the 2016 rainy season (autumn and winter). Twenty-one sampling sites in the lagoon were randomly selected and identified in the field by Global Positioning System (GPS) (Etrex Garmin make^®^). Sixteen (n = 16) water samples represented the agriculture zone, eight samples (n = 8) represented the industrial zone and eighteen samples (n = 18) represented communal lands. The samples were collected in a 1 L glass bottles using standard sampling procedures [31].

The levels of pH, water electrical conductivity (ECw), and temperature were determined in the field immediately after sampling using a multi-parameter (HI-98130 pH/CE/TDS/°C, HANNA Instruments, Woonsocket, RI, USA). The electrodes were properly rinsed with deionized water prior to every measurement to avoid contaminating the samples. The samples were stored on ice during transportation and then stored at 4 °C until being analyzed.

### 2.4. Sample Preparation and Extraction

Conventional methods for the extraction of organochlorine pesticides like DDT in water include: liquid-liquid extraction (LLE) and solid phase extraction (SPE) [32,33]. Although these methods are effective, the intensive work is tedious and time-consuming, and given the use of large amount of solvents, it is not environmental friendly.

Solid-phase micro extraction (SPME) is a solvent-free technique that is faster and simpler than conventional extraction methods. In this study, SPME was selected since it is a sensitive and robust extraction technique for organic compounds in aqueous matrices without the use of solvents, representing an environmentally friendly and efficient alternative for measuring micropollutants. SPME using a coated fiber with 100 µm of polydimethylsiloxane (PDMS) has been reported suitable for the trace analysis of persistent organic pollutants like DDTs with recovery rates of up to 100% [15,34,35].

In relation to ion methodologies, atomic absorption spectroscopy (AAS), inductively coupled plasma mass spectrometry (ICP-MS) and ion chromatography (IC) have been reported by other researchers for the analysis of metals and metalloids. However, IC is a powerful technique for water chemistry analysis that can measure concentrations of major anions, such as chloride, nitrate, nitrite, and sulfate, as well as major cations such as lithium, sodium, ammonium, potassium, calcium, and magnesium in the range of parts-per-billion (ppb) [36]. Anions were analyzed according to the EPA Method 300.0 (A) [37] and cations according to ASTM Method D6919-03 [38]. Both methods use ion chromatography.

The water samples were prepared by pouring 2900 µL of the sample and 100 µL of the internal standard at a final concentration of 30 ng/mL in a 10 mL headspace vial. Vials were sealed with aluminum caps furnished with Polytetrafluoroethylene-faced silicone septum and immersed in a sand bath to maintain a temperature of 100 °C. After 5 min of stabilization, the SPME fiber was exposed to agitation at 700 rpm in the headspace mode for 60 min. After the extraction step, the fiber was immediately desorbed into the injector of the Gas Chromatograph (GC) for pesticide analysis.

### 2.5. Instrumental Analysis

The concentrations of cations and anions were determined by ion chromatography using a Thermofisher Dionex ICS-1100 (Thermo Fisher Scientific, Waltham, MA, USA) equipped with a conductivity detector and an autoinjector (AS-DV). Anions were analyzed with an AS19 column (4 × 250 mm) and AERS 500 suppressor (4 mm). Cations were determined using a column CS12A, (4 × 250 mm) and a CERS 500 suppressor (4 mm).

DDT was analyzed with a gas chromatograph (Agilent Techologies7890b, Santa Clara, CA, USA) coupled to a mass spectrometer (Agilent Technologies 5975C, Santa Clara, CA, USA). The system was programmed in the electron impact mode (EI 70 eV). The separation was achieved with a 30 m × 0.25 mm I.D. TG-5HT column (Thermo Fisher Scientific, Swedesboro, NJ, USA) coated with 5% diphenyl/95% dimethyl polysiloxane (film thickness 0.25 µm). The samples were analyzed in the splitless mode. High-purity (99.99%) helium was employed as the carrying gas at a rate of 1 mL/min. The column temperature was programmed for 60 °C for 5 min at a rate of 8 °C/min until reaching 280 °C, which it held for 4 min. Mass spectrometer (MS) source and MS quad temperatures were set at 230 °C and 150 °C, respectively.

### 2.6. Quality Assurance and Quality Control (QA/QC)

For DDTs, quality control was evaluated on a five concentration level calibration curve (0.4, 0.8, 5, 10 and 50 ng/mL), blanks and duplicates per sample were included. The linear correlation coefficient (r2) for every analyte was 0.92 or higher for both the GC/MS and IC analytical methods. The relative standard deviations (RSD) of the duplicates per sample were < 20%, indicating acceptable levels of accuracy. The recovery percentage of the SPME samples were calculated by adding the internal standard (1-Bromine, 2-Nitrobenzene) to the water samples to obtain a final concentration of 30 ng/mL. The instrumental responses of the internal standard (area counts in the total ion chromatogram) from the water samples were compared to the instrumental response of internal standard at the same concentration in ultra-pure water. The recovery rates of DDT and its metabolites using SPME extraction were up to 93%.

### 2.7. Statistical Analysis

All statistical calculations were made with MiniTab 16^®^ (Minitab Inc., State College, PA, USA). Analysis of variance (ANOVA) was applied to pH, ECw, Na^+^ and Cl^−^, DDTs. All statistical analyses used a level of significance of 0.05 (α = 0.05).

## 3. Results

### 3.1. Physical-Chemical Parameters of the Water

**Figure 2:**
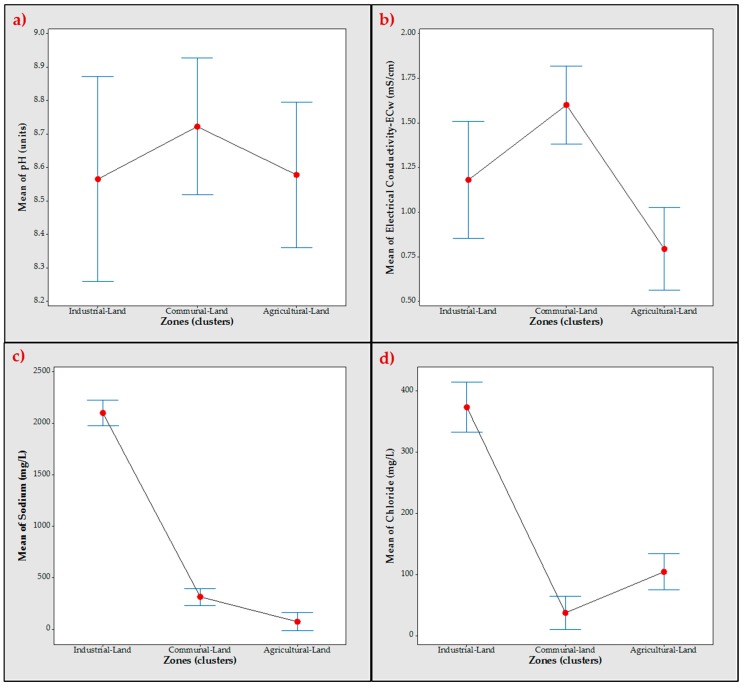
Levels of the parameters (**a**) pH, (**b**) ECw, (**c**) Na^+^ and (**d**) Cl^−^ in water samples obtained in Bustillos Lagoon in Chihuahua, Mexico.

The ANOVA did not detect statistically significant differences in pH levels among the zones (*p* > 0.05). The mean pH level in the area close to the agricultural zone was 8.57 ± 0.42 mg/L, in the industrial zone it was 8.56 ± 0.28 mg/L and in the communal zone was it 8.72 ± 0.05 mg/L. The ANOVA for water electrical conductivity showed statistically significant differences among the zones (*p* < 0.05). The average in the agricultural zone was 0.79 + 0.35 mg/L, in the industrial zone 1.18 ± 0.53 mg/L and the communal zone 1.60 ± 0.00 mg/L. Na^+^ levels varied among the zones (*p* < 0.05). The maximum level was 2099 ± 274 in the industrial zone, while in the communal zone it was 314.18 ± 4.83 and the minimum of 72.5 ± 56.3 was in the agriculture zone. The difference among the zones in Cl^−^ was statistically significant (*p* < 0.05). The mean of Cl^−^ was highest in the industrial zone at 373.6 ± 60, while in communal zone it was 37.27 ± 1.20 and in the agriculture zone it was 104.4 ± 49.3. These results are shown in Figure 2.

Table 1 presents the parameters in Bustillos Lagoon and the usual concentrations recommended by the FAO for irrigation and livestock drinking water.

### 3.2. Concentrations and Occurrence of DDT in Water

This study analyzed DDT degradation products: 2,4′-DDE, 4,4′-DDE, 2,4′-DDD, 2,4′-DDT, 4,4′-DDD, 4,4′-DDT. The six analytes were detected in all water samples. The ANOVA detected statistical differences among the zones for DDT (*p* < 0.05). In general, the highest concentrations in Bustillos Lagoon were in zone 3, which is the closest site to agricultural activities. In this zone, the total average concentration of all the analytes was ∑DDTs _Total_ = 811 ng/mL. The most abundant isomers were DDT > DDD > DDE. Zones 1 and 2 had similar concentrations of all the metabolites, with concentrations of 7.21 ng/mL in zone 1 and 7.42 ng/L in zone 2 shown in Table 2.

**Figure 3:**
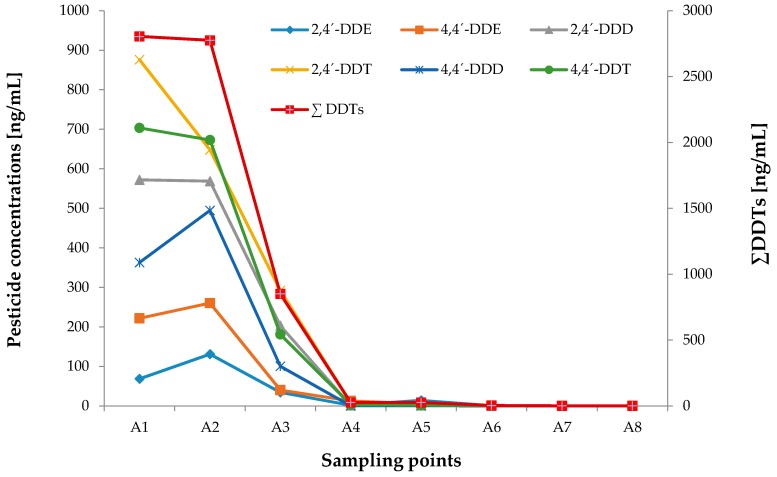
Distribution and concentrations of DDT and its metabolites in zone 3.

The high levels of DDTs detected in zone 3 may be associated with wind transport or agricultural irrigation runoff. Zone 3 features the inflow to the lagoon of the stream El Muerto, which expose the lagoon to irrigation runoff from all agricultural activities in the area. Interestingly, the concentrations were 99.8% lower at sampling sites A4 to A8, which could be due to dilution in the lagoon (Figure 3).

## 4. Discussion

Generally the pH levels in the lagoon were slightly alkaline (8.62 ± 0.07) and exceeded the levels recommended by the FAO for irrigation (6.5–8.5). The highest pH value was observed in zone 3 where two samples (A3 and A6) reported pH ≥ 9.

High salt content in irrigation water can cause similar problems as those produced by drought, resulting in reduced crop yield [4]. High concentrations of sodium (Na^+^) and chloride (Cl^−^) in irrigation water affect sensitive crops. These ions are concentrated in soil as water is lost through evaporation. Cl^−^ is one of the most harmful ions in irrigation water. If it is not properly absorbed by the soil, it is absorbed by plants and accumulates in leaves, causing leaf burn or drying of leaf tissue.

Zone 1 presented the highest levels of Na^+^ and Cl^−^ ions, with concentrations around 2361 mg/L. and 453 mg/L, respectively. This zone reported Na^+^ concentrations that were more than 2.5 times as high as the FAO guideline, while Cl^−^ concentrations in all zones were below the usual range. The most heterogenic distribution of Na^+^ and Cl^−^ was observed in zone 3, the concentrations of Na^+^ were from 9 to 158 mg/L, while the levels of Cl^−^ were in the range of 49 to 165 mg/L. The concentrations were in the normal range according to the FAO. The highest sodium adsorption ratio (SAR) values were reported in zone 1, with a range of 36 to 41 meq/L. The usual range of this parameter for irrigation is 15 meq/L. SAR was also calculated in zone 3, where the values ranged from 0.06 to 1.1 meq/L. Zone 2 was the only area where calcium (Ca^2+^) and magnesium (Mg^2+^) were not detected, thus SAR could not be determined. SAR is a measure of water quality for agricultural irrigation, such that the higher the SAR, the greater hazard posed by sodium. The elevated SAR values can be attributed to ongoing industrial wastewater discharges since 1952 [39].

The water electrical conductivity throughout the study area was in the range of 0.27 to 1.45 mS/cm. Based on FAO criteria, these values indicate a slight to moderate risk (0.7–3 mS/cm) for crops irrigated with this water.

The nitrate (NO_3_^−^) ion was only detected in zone 3, with values ranging from <0.42 to 59 mg/L, which are above the maximum of 10 mg/L recommended by the FAO. Sulfate (SO_4_^2−^) was only detected in zones 1 and 3, with levels in zone below the limit recommended by the FAO for sensitive crops.

In relation to livestock drinking water, ECw does not represent a risk for animals. The National Academy of Sciences has stated that livestock drinking water with an ECw of less than 5 mS/cm is safe under almost any circumstance [4,40].

Elevated concentrations of Mg^2+^ can cause scouring and diarrhea in livestock. The FAO recommends a limit of 250 mg/L for lactating cows and 400 mg/L for beef cattle. The level of Mg^2+^ in drinking water for lactating cows in zone 3 exceeded this limit, with concentrations in the range of 270 to 279 mg/L. The concentrations of nitrates (NO_3_^−^) and nitrites (NO_2_^−^) are considered when evaluating water quality for livestock. The FAO guideline established an upper limit of 100 mg/L for the sum of nitrates and nitrites (NO_3_^−^ + NO_2_^−^), a level that was not found in any of the three analyzed zones [4].

Considering all the physical and chemical parameters measured, only the water in zone 2 would be acceptable for use for irrigating plants with high tolerance to stress but not for crops or livestock consumption. The water in zones 1 and 3 can represent a risk to sensitive crops and the health of livestock.

Previous studies of lagoon systems in Mexico have reported a variety of organochlorine pesticides, DDT most often being predominant among them. The levels of total DDTs reported in Laguna de Terminos in the State of Campeche were ∑DDTs_Total_ = 0.00029 ng/mL [15]. DDD residues have also been reported in waters from Pozuelos–Murillo Lagoon in southern Mexico, with concentrations of 2 ng/mL. The levels reported in these two studies were much lower than those found in this study. The levels of ∑DDT < 1000 ng/mL in zones 1 and 2 reach the maximum Mexican regulatory limits for drinking water [41]. Only two sampling sites from zone 3 (A1 = 2804 ng/mL, A3 = 2775 ng/mL) were almost 3 times as high as the limits for drinking water according to Mexico regulations (Figure 3). The levels of 4,4’-DDE in zone 3 of this study were almost 20 times as high as the limit established by Mexican federal law (4 ng/mL) for irrigation water in agriculture.

Total DDTs in a typical agricultural area of China range from 0.00442 to 0.269 ng/mL in surface water [42]. Feng et al., 2013 reported DDT levels in the range of 0.2 to 9.3 ng/mL in the water of the Yangtze River Delta, China, which is used for rice production [43]. Fu et al., 2003 reported concentrations up to 1.2 ng/mL in the waters from Guangzhou, China [44]. In 2016, the concentration of 2,4′-DDT in water used to irrigate rice crops was 228.8 ng/mL in Guamo, Colombia [45]. In Argentina, the concentrations of DDTs detected in surface water of two aquifers ranged from 3 to 5.40 ng/mL [46]. In Bangladesh, DDT and DDE were detected in irrigated water at concentrations of 4.06 to 8.29 ng/mL, respectively [47].

DDT concentrations reported in all these studies are comparable to concentrations in zones 1 and 2, only the concentrations in zone 3 sampling sites A1 and A3 were significantly higher than those reported in surface water. Concentrations of 2,4’-DDT detected in Colombia were very similar to those in zone 3 of this study. To the authors’ knowledge, this is the first study of DDT and its metabolites in an endorheic lagoon.

## 5. Conclusions

Bustillos Lagoon is recognized as one of the most important water bodies in North America. In a semi-arid region like Chihuahua, Bustillos Lagoon represents a valuable source of water for irrigation and livestock production.

The results of this study indicate that the water in zones 1 and 3 represent a risk for crop irrigation and as livestock drinking water due to the high levels of SAR, nitrates and magnesium. In addition, DDT concentrations in Bustillos Lagoon are much higher than those found in other studies of Mexican water bodies. The highest concentrations of DDT and its metabolites were found in zone 3, which indicates the transports of pesticides, including DDTs, in irrigation runoff.

The water from zone 2 is the least contaminated. Nevertheless, its use can only be recommended for irrigating stress tolerant plants and not for crops. It is highly recommended to treat water used for any purpose to minimize adverse effects on the environment and animal health.

Organochlorine pesticides like DDT tend to bioaccumulate and biomagnify in the food chain. DDT and its metabolites have been detected in beef and dairy products, which means that humans are exposed through food consumption. Other routes of DDT exposure for cattle are contaminated feed, grass and water.

## Figures and Tables

**Table 1:** Parameters to evaluate common irrigation water quality problems and levels detected in the study area. nd = not detected.

	Water Quality for Irrigation			FAO
Parameter	Zone 1	Zone 2	Zone 3	Usual Range in Irrigation Water
pH	7.8–8.98	8.65–8.84	8.2–9.3	6.5–8.5
Na^+^ (mg/L)	1678–2361	305–321	9–158	920
Cl^−^ (mg/L)	326–453	34–38	49–165	1065
SAR (meq/L)	36–41	nd	0.06–1.1	0–15
NO_3_^−^ (mg/L)	<0.42	<0.42	<0.42–59	0–10
SO_4_^2−^ (mg/L)	233–932	<2.085	40–136	960
ECw (mS/cm)	0.38–1.45	1.6	0.27–1.21	0–3
	Drinking water for livestock			Desired Level
Mg^2+^ (mg/L)	<0.0191	<0.0191	270–279	250 cows (lactacting) 400 beef cattle
NO_3_^−^ + NO_2_^−^ (mg/L)	<0.78	<0.78	<0.78–71	100

**Table 2:** Average concentrations (±standard deviation) and distribution of DDTs in the study area.

DDT Metabolites	Concentration ng/mL		
	Zone 1	Zone 2	Zone 3
2,4′-DDE	1.40 ± 0.014	1.44 ± 0.030	31.3 ± 46.81
4,4′-DDE	1.13 ± 0.003	1.18 ± 0.058	67.7 ±108.23
2,4′-DDD	0.77 ± 0.003	0.80 ± 0.035	168.2 ± 257.72
2,4′-DDT	0.70 ± 0.004	0.75 ± 0.050	228.6 ± 349.43
4,4′-DDD	1.55 ± 0.002	1.57 ± 0.018	119.8 ± 196.97
4,4′-DDT	1.66 ± 0.001	1.68 ± 0.016	195.4± 310.50
Total DDTs	7.21	7.42	811
